# Flagella from Five *Cronobacter* Species Induce Pro-Inflammatory Cytokines in Macrophage Derivatives from Human Monocytes

**DOI:** 10.1371/journal.pone.0052091

**Published:** 2012-12-21

**Authors:** Ariadnna Cruz-Córdova, Luz M. Rocha-Ramírez, Sara A. Ochoa, Bertha Gónzalez-Pedrajo, Norma Espinosa, Carlos Eslava, Ulises Hernández-Chiñas, Guillermo Mendoza-Hernández, Alejandra Rodríguez-Leviz, Pedro Valencia-Mayoral, Stanislaw Sadowinski-Pine, Rigoberto Hernández-Castro, Iris Estrada-García, Onofre Muñoz-Hernández, Irma Rosas, Juan Xicohtencatl-Cortes

**Affiliations:** 1 Laboratorio de Bacteriología Intestinal, Departamento de Infectología, Hospital Infantil de México Federico Gómez, México D.F., México; 2 Departamento de Genética Molecular. Instituto de Fisiología Celular, Universidad Nacional Autónoma de México, México, D.F., México; 3 Departamento de Salud Pública, Facultad de Medicina, Universidad Nacional Autónoma de México, México, México, D.F., México; 4 Departamento de Bioquímica, Facultad de Medicina, Universidad Nacional Autónoma de México, México, D.F., México; 5 Departamento de Patología. Hospital Infantil de México Federico Gómez. Dr. Márquez 162, Col. Doctores, Delegación Cuauhtémoc, México D.F., México; 6 Departamento de Ecología de Agentes Patógenos. Hospital General “Dr. Manuel Gea González”, Tlalpan, México; 7 Centro de Ciencias de la Atmósfera, Universidad Nacional Autónoma de México, México, D.F., México; 8 Departamento de Inmunología Molecular, Escuela Nacional de Ciencias Biológicas, IPN. Unidad Profesional Prolongación, Miguel Hidalgo, D. F., México; University of São Paulo, Brazil

## Abstract

*Cronobacter* spp. are opportunistic pathogens linked to lie-threatening infections in neonates and contaminated powdered infant formula that has been epidemiologically associated with these cases. Clinical symptoms of *Cronobacter* include necrotizing enterocolitis, bacteremia, and meningitis. Flagella from *C. sakazakii* are involved in biofilm formation and its adhesion to epithelial cells. We investigated the role of flagella from *C. sakazakii* ST1 and ST4, *C. malonaticus, C. muytjensii, C. turicensis* and *C. dublinensis* during the activation of cytokines (IL-8, TNF-α, and IL-10) in macrophage derivatives from human monocytes, which has not been extensively studied. The production and identity of flagella from the five *Cronobacter* species were visualized and recognized with anti-flagella antibodies by immunogold labeling through transmission electron microscopy. Purified flagella were dissociated into monomers in 12% SDS-PAGE Coomassie blue-stained gels showing a band of ∼28 kDa and, in addition, mass spectrometry revealed the presence of several peptides that correspond to flagellin. Flagella (100 ng) induced the release of IL-8 (3314–6025 pg/ml), TNF-α (39–359 pg/ml), and IL-10 (2–96 pg/ml), in macrophage isolates from human monocytes and similar results were obtained when flagella were dissociated into monomers. Inhibition assays using three dilutions of anti-flagella antibodies (1∶10, 1∶100, and 1∶200) suppressed the secretion of IL-8, TNF-α, and IL-10 between 95–100% using 100 ng of protein. A transfection assay using 293-hTLR5 cells showed IL-8 release of 197 pg/ml and suppression in the secretion of IL-8 when anti-hTLR5-IgA antibodies were used at different concentrations. These observations suggest that flagella and flagellin are involved in an inflammatory response dependent on TLR5 recognition, which could contribute to the pathogenesis of the bacteria.

## Introduction

*Cronobacter* spp. (formerly *Enterobacter sakazakii*) are facultative, Gram-negative, non-spore forming, motile microorganisms that belongs to the *Gammaproteobacteria* class and to the Enterobacteriaceae family [1]. Currently, based on the phenotypic and genotypic characterization of this genus, seven species have been described: *Cronobacter sakazakii*, *C*. *malonaticus*, *C*. *dublinensis*, *C*. *muytjensii*, *C. turicensis*, *C. universalis*, and *C. condimenti* [2], [3], [4], [5], [6]. *Cronobacter* is an ubiquitous organism that can be isolated from a wide range of environments, including water, soil, vacuum cleaner dust, air samples, rhizosphere, and a variety of processed foods and fresh produce [7], [8], [9]. The mechanisms of transmission of these bacteria have been associated with the ingestion of contaminated reconstituted formula, but it has also been isolated from a variety of foods (from animal and vegetable origin) [8], [10].

Identification among *Cronobacter* species is difficult due to the diversity of the genus. A Multi Locus Sequence Typing (MLST) of seven housekeeping genes was originally developed for the differentiation between *C. sakazakii* and *C. malonaticus*, two species that could not be distinguished according to the 16S rDNA [11]. This molecular tool has provided an effective typing scheme for the *Cronobacter* genus, showing a high level of discernment between the isolates. Interestingly, MLST has identified *C. sakazakii* ST4 as the predominant sequence type isolated from cerebral spinal fluid from meningitis cases [12].

*Cronobacter* species are considered opportunistic pathogens that have been implicated in life threatening diseases in humans, across all group ages [13]. However, particularly neonates of low-birth weight are the major risk group identified with a high mortality rate (40–80%) [14]. This pathogen is a rare cause of neonatal meningitis, septicemia, and necrotizing enterocolitis in infants [15].

Although several genes have been identified to be involved in the virulence of *Cronobacter* species, we are still far from understanding their pathogenesis. On the other hand, not all *Cronobacter* species has been linked with infections and the severity of virulence varies among strains. *Cronobacter* species vary in their virulence with respect to the invasion of intestinal cells, enterotoxin production, survival in macrophages, and serum resistance [16], [17], [18], [19]. Recently, it has been suggested that the outer membrane proteins OmpA and OmpX from *C. sakazakii* are involved in basolateral invasion of human enterocyte-like Caco-2 and intestinal INT407 epithelial cells [19], [20], [21]. These data are the first report of *C. sakazakii* virulence determinants essential for invasion that may be critical for the pathogenicity of this microorganism. Other studies showed the ability of *Cronobacter* spp. to adhere to two epithelial cell lines (HEp-2 and Caco-2 cells), as well as to a brain microvascular endothelial cell line [17]. In addition, *C. sakazakii* utilizes dendritic cells (DCs) as a vehicle for propagation and survival, hence evading potential immune surveillance [22]. Recently, the role of PMNs (polymorphonuclear leukocytes) and macrophages was examined in acute *C. sakazakii* induced mouse model of NEC (necrotizing enterocolitis). Oral feeding of *C*. *sakazakii* results in acute intestinal inflammation and death in newborn mouse pups; the presence and recruitment of PMNs and macrophages to the lamina propria is important for clearance of the bacteria during initial states of the infection. Furthermore, their absence exacerbates mucosal injury by increasing the levels of pro-inflammatory cytokines [23]. *Cronobacter* spp. are also involved in biofilm formation on glass, stainless steel, polyvinyl chloride, polycarbonate, silicone, and enteral feeding tubes which could represent the vehicle of infection [24], [25]. The survival of *C. sakazakii* in biofilms is due to the presence of multiple nutritional factors and/or different environmental conditions, which is associated with the increase in antibiotic resistance [26], [27]. In other pathogens, biofilm formation is highly dependent on the medium and surface used. Cellulose has been described as other component of the *Cronobacter* extracellular matrix that contributes to the formation of biofilm [28], [29], [30], [31]. *Cronobacter* spp. have been described as motile pathogens due to the presence of flagella [29]. Recent data showed that flagella of *C. sakazakii* are involved in biofilm formation in abiotic surfaces and contribute in the adhesion to Caco-2 epithelial cells [29].

Flagella of many Gram-negative pathogens are capable of activating the release of pro-inflammatory cytokines in epithelial, monocytic, polymorphonuclear, and dendritic cells [32]. Recognition of flagellins of many bacterial pathogens via Toll-like receptor 5 (TLR5) triggers the activation of cytokines such as IL-8, IL-1β, tumor necrosis factor-α (TNF-α), and IL-6 [33]. Purified flagella and secreted flagellins (FlaA, FlaC, and FlaD) from *Vibrio cholerae* in cell-free supernatants are involved in the induction of IL-8 in epithelial cells [34]. However, to have a better understanding of the role of flagella during colonization of the host, it is necessary to perform other studies that allow us to know more about its function as a virulence factor. The role of *Cronobacter* flagella and flagellin in the activation of pro-inflammatory cytokines in macrophages has not been explored. We investigated the participation of these proteins produced by *C. sakazakii* ST1, *C. sakazakii* ST4, *C. malonaticus, C. muytjensii, C. turicensis* and *C. dublinensis* in the release of several pro-inflammatory and anti-inflammatory cytokines, mainly IL-8, TNF-α, and IL-10 in macrophages derived from human monocytes; in addition, we will determine if this inflammatory response is dependent on TLR5 recognition.

## Results

### Biochemical Analysis of Flagella

**Figure 1:**
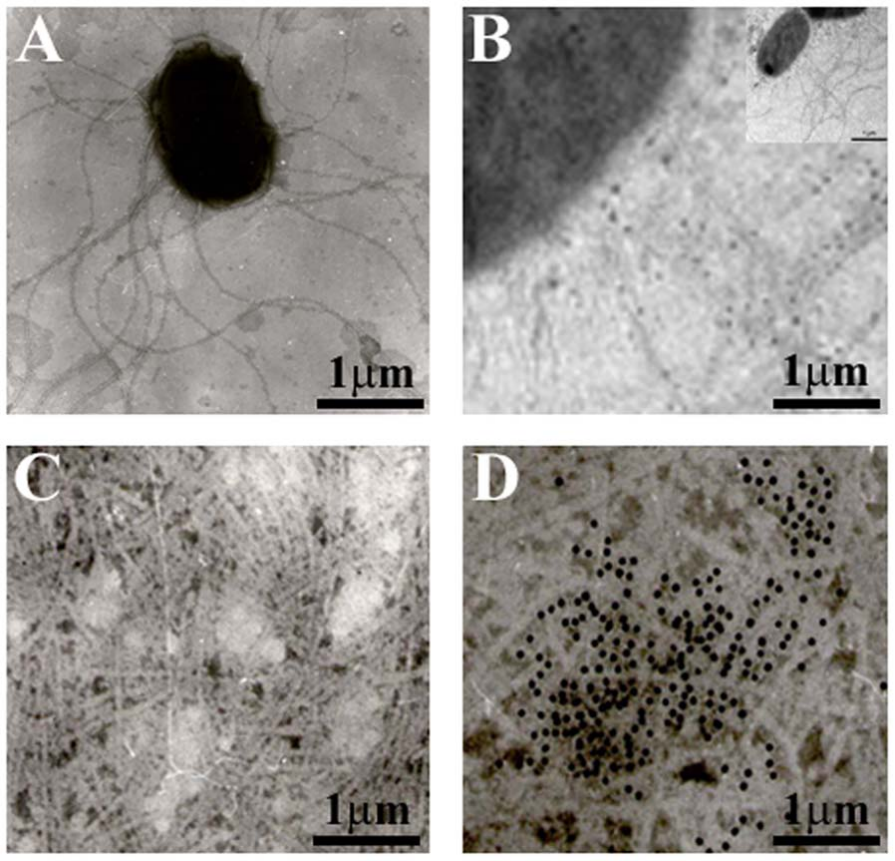
Flagella identification by transmission electron microscopy. (A) *C. sakazakii* ATCC BAA-894 strain shows flagellar structures protruding from the bacteria after growth on TSA agar at 37°C. (B) Immunogold-labeling of flagella produced by *C. sakazakii* on TSA agar using anti-flagella antibody. The inset shows the micrograph of the whole bacteria using immunogold-labeling obtained by TEM. (C) Purified flagella. (D) Immunogold-labeling of purified flagella. Samples were negatively stained and electron micrographs were taken at a magnification of 19,000x.

The flagella preparation was rich in flagellar filaments as determined by negative staining and electron microscopy. Images obtained by TEM demonstrated the presence of flagellar structures with filaments of ∼3.1 µm long and 0.06 µm width (Fig. 1A). Flagella protruding from the bacterial cells were labeled with gold particles using anti-flagella polyclonal antibodies, confirming the identity of these structures (Fig. 1B). In this figure, gold particles are interacting around the flagella and not with the bacteria showing the specificity of the antibodies. To further determine the nature of these structures, purification of flagella by mechanical shearing and differential centrifugation was performed. Purified flagella from *C. sakazakii* were visualized by TEM and their identity was detected by immunogold labeling using anti-flagella antibody (Fig. 1C and D). Immunoblotting and TEM assays showed no reaction against purified flagella with the preimmune serum, which was used as a negative control (data not shown).

**Figure 2:**
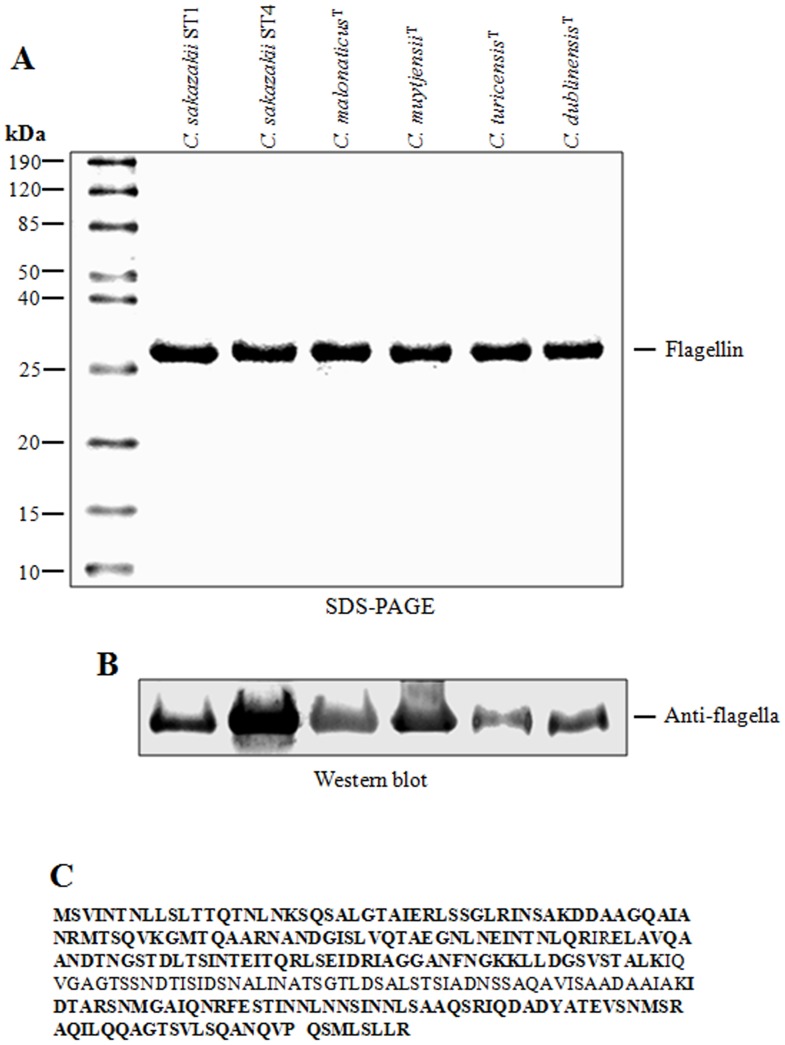
Biochemical analysis of flagella. (A) Depolymerization of purified flagella in 12% SDS-PAGE gel. (B) Immunoblot assays with anti-flagella antibody recognizing the flagellin of ∼28-kDa. (C) Analysis of the protein band of ∼28-kDa by mass spectrometry. Molecular weight (MW).

Purified flagella were dissociated into flagellin monomers with a molecular mass of ∼28 kDa, which were visualized in 12% SDS-PAGE Coomassie blue-stained gels (Fig. 2A). In addition, results obtained by immunoblotting confirmed the identity of the excised ∼28-kDa putative flagellin of *C. sakazakii* (ST1 and ST4), *C. malonaticus, C. muytjensii, C. turicensis*, and *C. dublinensis* when reacted with the polyclonal antibody (Fig. 2A). Mass spectrometry analysis of the ∼28-kDa protein band for each of the species analyzed revealed the presence of several peptides corresponding to the amino acid sequence of the hypothetical protein encoded by the ESA_01288 gene from *C*. *sakazakii* ATCC BAA-894. A high identity (99%) was observed among the protein encoded by the ESA_01288 and *fliC* genes from *C*. *turicensis*. We propose the generic name FliC for the protein encoded by the ESA_01288 gene herein *fliC* (Fig. 2B).

### Sequence Analysis of *Cronobacter* Species Flagellin

**Figure 3:**
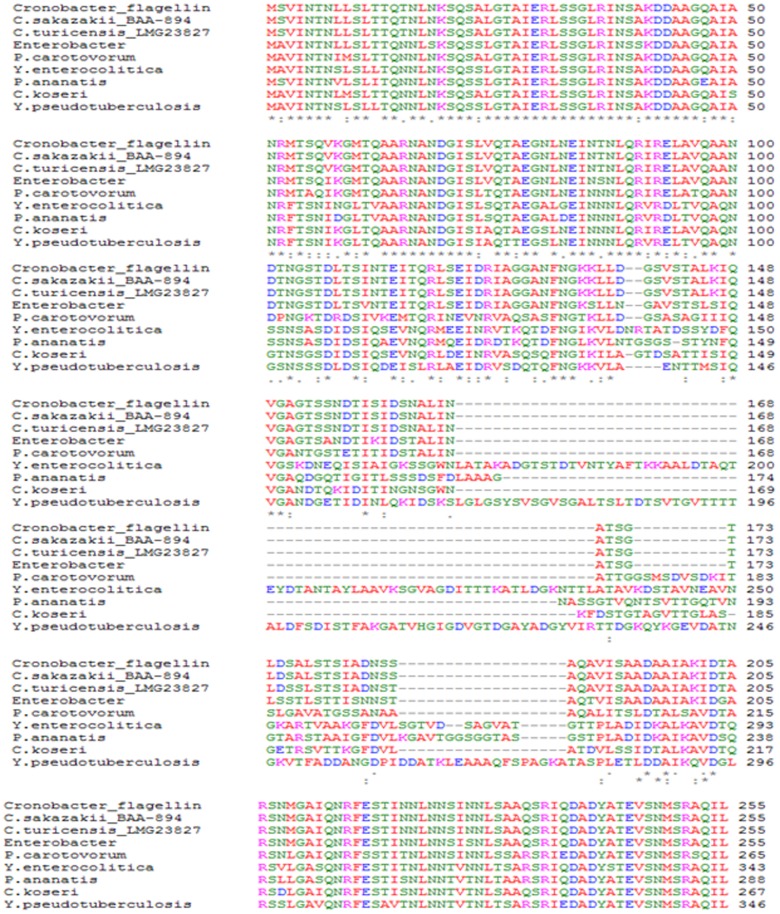
Multiple sequence alignment of flagellin from *C. sakazakii* ST1 ATCC BAA-894, *C. sakazakii* ST4 ATCC 29004, *C. malonaticus^T^, C. muytjensii^T^, C. turicensis^T^*, and *C. dublinensis* ^T^ (*Cronobacter_*flagellin) with different known Gram-negative bacterial flagellins. Sequences were aligned using the ClustalW progam. *C. sakazakii*, *C turicensis, Enterobacter* spp., *Pectobacterium carotovorum* subsp. *Carotovorum*, *Citrobacter koseri* ATCC BAA-895, *Yersinia enterocolitica*, *Pantoea ananatis* LMG 20103, and *Yersinia pseudotuberculosis* IP 32953.

The identity analyses were performed to evaluate if any common epitopes were shared among different flagellins. Results obtained after digestion of the polypeptide and N-terminal amino acid sequence analysis, showed that the ∼28-kDa protein is in fact the flagellin structural protein, which is highly identical to other bacterial flagellins. Multiple amino acid sequence alignments showed that the primary sequence of FliC from *Cronobacter* species is highly similar to flagellins from *C*. *turicensis* (99% identity), *Enterobacter* spp. (90%), *Pectobacterium carotovorum* subsp. *carotovorum* (76%), *Citrobacter koseri* ATCC BAA-895 (68%), *Yersinia enterocolitica* (62%), *Pantoea ananatis* LMG 20103 (62%) and *Yersinia pseudotuberculosis* IP 32953 (61%) (Fig. 3); other pathogens showed a lower level of identity, like the flagellin of *Escherichia coli* (50%) (data not shown).

### Flagella are Involved in the Release of Pro-inflammatory Cytokines

Lipopolysaccharides, flagella, and pili of several bacterial pathogens have been considered as activators of the secretion of a variety of pro- and anti-inflammatory cytokines [20], [36]. We investigated the potential role of flagella from *Cronobacter* species as an inducer of pro-inflammatory (IL-8 and TNF-α) or anti-inflammatory (IL-10) cytokines from macrophages isolated from human monocytes. Endotoxin from flagella in *Cronobacter* species was removed using polymixin B agarose (Sigma, St Louis CA) according to manufacturer’s recommendation. Macrophage cells were incubated with purified flagella for 12, and 24 h at 37°C, statically, and under a 5% CO_2_ atmosphere. In this study, we purified flagella from five *Cronobacter* species: *C. malonaticus, C. muytjensii, C. turicensis*, *C. dublinensis*, and two *C. sakazakii* ATCC strains identified as ST1 (strain number: 658) and ST4 (strain number: 470) described by Baldwin *et al*. ST1 was selected because it was the first *C. sakazakii* genome described and ST4 is the most prevalent sequence type showing a correlation with meningitis infections [11]. These data are available online at www.pubmlst.org/Cronobacter.

**Figure 4:**
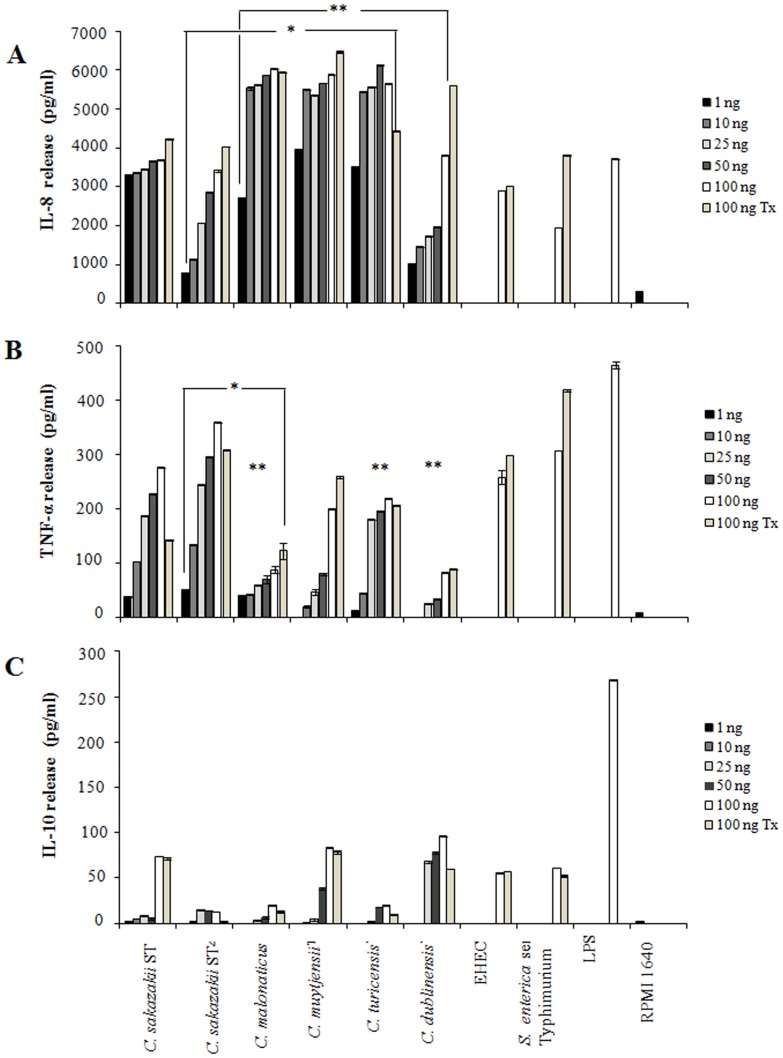
Flagella from *C. sakazakii* activate cytokine’s release. Macrophage cells were exposed to flagella (1, 10, 25, 50 and 100 ng/ml) and flagellin (100 ng/ml Tx) for 24 h. (A) High values were observed in the IL-8 release due to the presence of different concentrations of purified flagella from *Cronobacter* species. (B) Induction of TNF-α in macrophage cells in response to different concentrations of purified flagella from *Cronobacter* species. (C) A non-significant release of IL-10 was observed when two different concentrations of purified flagella from *Cronobacter* species were incubated. In addition, cytokines release was also observed when the purified flagella of each *Cronobacter* species were dissociated in monomers by heat. Lipopolysaccharide 100 ng/ml (LPS), RPMI-1640 medium. Flagellin of *Cronobacter* (Tx). Flagella of EHEC strain O157:H7 and Flagella from *S. enterica* serovar Typhimurium (100 ng/ml). * and ** *p<*0.05.

Cytokine release (IL-8, TNF-α, and IL-10) was measured at 12 and 24 h, and no differences were observed. A linear increase in IL-8, TNF-α, and IL-10 release was observed when different concentrations (1, 10, 25, 50 and 100 ng/ml) of flagella and flagellin (100 ng/ml) from *C. sakazakii* (ST1 and ST4), *C. malonaticus, C. muytjensii, C. turicensis*, and *C. dublinensis* were tested (Fig. 4A, B and C). These results confirmed that the levels induced during the activation of the three cytokines are dose-dependent of the protein concentration. As expected, high levels of released IL-8 were detected when flagella were tested under the same concentrations used for TNF-α (Fig. 4A and B). In sum, cell-free supernatants obtained from macrophage cells incubated with flagella at different concentrations showed levels of 3314 to 6025 pg/ml of IL-8 (*P*<0.05) and 39 to 359 pg/ml of TNF-α (*P*<0.05), respectively (Fig. 4A and B). However, when IL-10 was tested in the supernatant by ELISA a low concentration of 1.6 to 96 pg/ml was detected (Fig. 4C). In all cases, flagella from enterohemorrhagic *Escherichia coli* O157:H7 (EHEC) and *Salmonella enterica* serovar Typhimurium were used as positive controls. FliC from EHEC showed values of IL-8, TNF-α, and IL-10 releases of 3014, 297, and 56 pg/ml, respectively; whereas, 3817, 417, and 52 pg/ml for FliC from *S. enterica* serovar Typhimurium were obtained, respectively.

Significant differences (p<0.05) were observed in IL-8 release among *C. sakazakii* ST4 and *C. malonaticus*; *C*. *muytjensii* and *C. turicensis*; *C. dublinensis* and *C. malonaticus, C*. *muytjensii* and *C. turicensis.* However, significant differences were not observed when compared between *C. sakazakii* ST1 and *C. sakazakii* ST4. For TNF-α, only significant differences (p<0.05) where shown when comparing *C. sakazakii* ST4 and *C. maloniticus*; *C. maloniticus*, *C. turicensis*, and *C. dublinensis*. No significant differences (p>0.05) were observed during IL-10 release.

We also found that levels of induction of TNF-α and IL-8 in macrophage cells incubated with pre-heated flagella (monomeric flagellin), were similar to the release of these cytokines by non-heated flagella (Fig. 4A and B). Boiling is required to dissociate the flagella into flagellin monomers, which are the actual active pro-inflammatory molecules [33]. These data support the role of FliC from *Cronobacter* species analyzed which could be considered as a potent inducer of pro-inflammatory cytokines.

### Inactivation of Pro-inflammatory Cytokines

**Figure 5:**
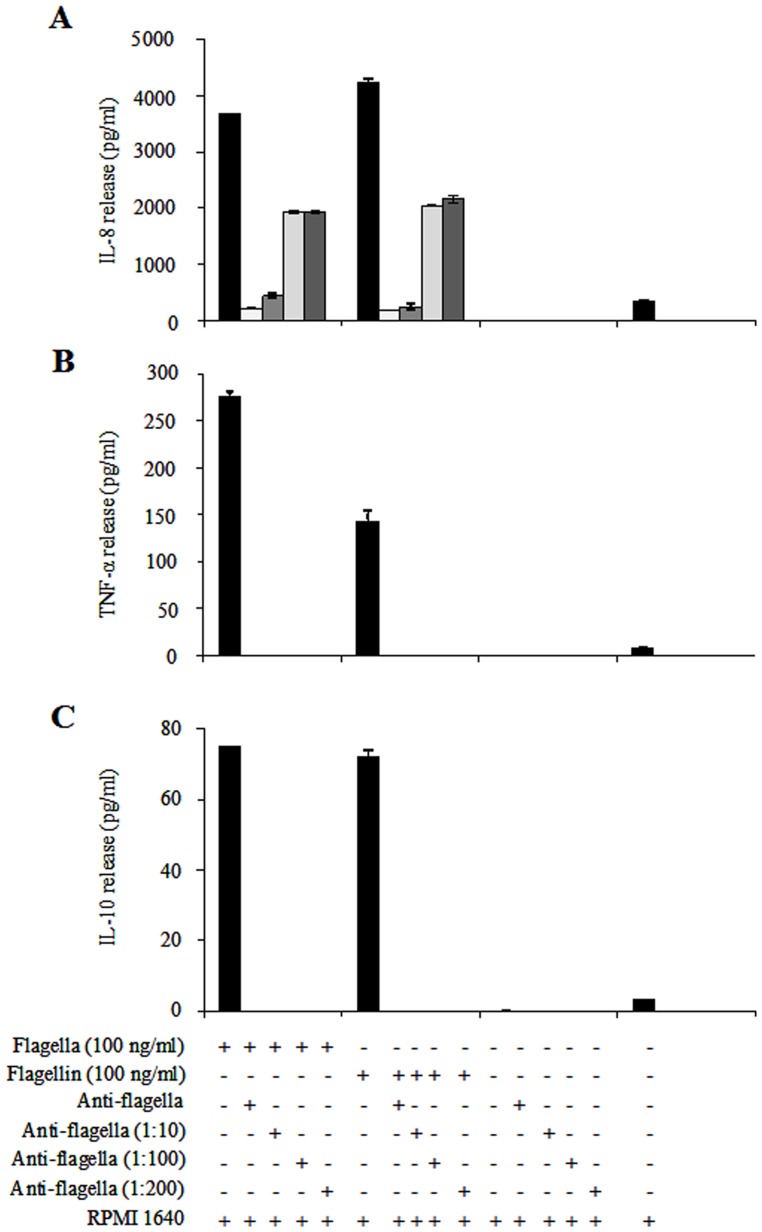
Anti-flagella antibodies block the secretion of pro-inflammatory cytokines. Purified flagella (100 pg/ml) and flagellin (100 ng/ml) were pre-incubated with anti-flagella antibodies in LPS-free water before the addition to macrophage cells. The antibodies were tested undiluted and using different dilutions (1∶10, 1∶100, and 1∶200). (A) IL-8 release was inhibited when flagella (94, 88, 48 and 48%) and flagellin (96, 94, 52 and 51%) were incubated with different dilutions of anti-flagella. (B and C) TNF-α and IL-10 releases were blocked completely with the three dilutions and with the undiluted antibody. RPMI-1640 medium.

To confirm the role of flagella and flagellin of *C. sakazakii* ST1 in the activation of pro- and anti-inflammatory cytokines (IL-8, TNF-α, and IL- 10), the purified proteins (100 ng/ml) were incubated for 1 h with undiluted and three different dilutions 1∶10, 1∶100, and 1∶200 of anti-flagella antibodies. The results showed a dose-dependent inhibition of IL-8 stimulation in macrophage monocyte cells after 24 h incubation (Fig. 5A). A maximum inhibition of 94% was observed when the undiluted antibody was used. The ability to block the release of IL-8 was affected by the three antibody dilutions employed, observing a significant loss in their capacity to inhibit the activation of this cytokine (Fig. 5A). Under the same conditions, the measured levels of TNF-α and IL-10, showed a complete inhibition when undiluted or diluted anti-flagella antibodies were employed (Fig. 5B and C). The antibody was used as a negative control to exclude any effect in the activation of IL-8, TNF-α, and IL-10 mediated by the presence of flagella (Fig. 5A, B and C). Likewise, the inhibition effects did not show differences with the other purified flagellins when tested in other species (data not shown).

### Effects of Anti-humanTLR5-IgA Antibody on *C. sakazakii* Flagella

**Figure 6:**
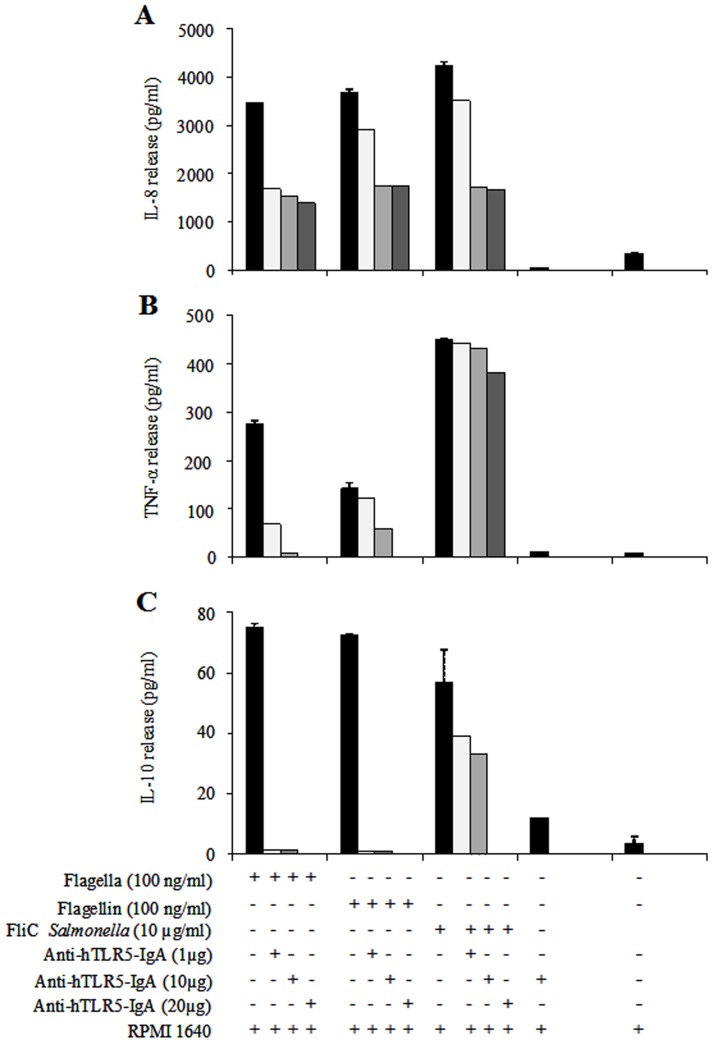
Anti-human-TLR5-IgA antibody blocked the secretion of pro-inflammatory cytokines. (A) An inhibition dependent on concentration of anti-hTLR5-IgA was observed in IL-8 release, in flagella (51, 55, and 59%) and flagellin (21, 52 and 53%) with 1, 10 and 20 µg of antibody, respectively. (B) TNF-α release was blocked between 75–97% for flagella and 13–58% for flagellin with 1 and 10 µg anti-human-IgA-TLR5, respectively. (C) IL-10 release was blocked 98% for flagella and 99% for flagellin with 1 and 10 µg anti-human-IgA-TLR5, respectively. RPMI-1640 medium.

To determine the role of flagellin-TLR5 recognition in the initiation of innate inflammatory responses in monocyte-derivatives of human macrophages, anti-hTLR5-IgA antibody was used at different concentrations (1, 10, and 20 µg/ml) to block the induction of cytokines released by flagella and flagellin (100 ng/ml) from *C. sakazakii* ST1. A dependent-inhibition was observed for IL-8, TNF-α and IL-10 in the three concentrations of anti-hTLR5-IgA tested (Fig. 6A, B and C). IL-8 release in the three concentrations of anti-hTLR5-IgA tested showed a reduction in cytokine activation of 51% with 1 µg, 55% with 10 µg, and 59% with 20 µg of anti-human-IgA-TLR5 (Fig. 6A). Similar results were obtained for the levels of IL-8 induction with flagellin; a reduction of 21% in IL-8 release with 1 µg, and 53% with 10 and 20 µg of anti-hTLR5-IgA was observed (Fig. 6A). Total inhibition was identified in the release of TNF-α and IL-10 when 20 µg/ml of anti-hTLR5-IgA were used. When anti-hTLR5-IgA was employed at 1 and 10 µg/ml, the activation of both cytokines was not completely abolished (Fig. 6B and C). The same effect was observed when other *Cronobacter* species were tested (data not shown). FliC from *S*. *enterica* serovar Typhimurium was used as the positive control.

### Flagella of *C. sakazakii* Induce IL-8 Release in 293-hTLR5 Cells

**Figure 7:**
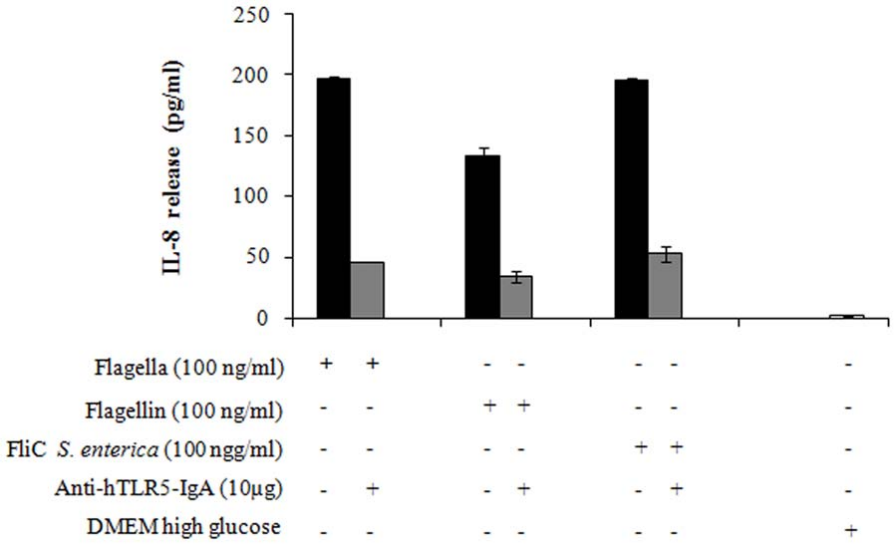
Transfection assay in HEK293-hTLR5 cells enables IL-8 secretion in response to *C. sakazakii* flagella and flagellin. 293-hTLR5 cells (HEK293 cells transfected with TLR5) secreted 197 pg/ml and 133 pg/ml of IL-8 after treatment with *C. sakazakii* (ST1) flagella and flagellin, respectively; and 196 pg/ml of IL-8 were released with FliC-*Salmonella enterica* serovar Typhimurium. 293-hTLR5 cells pre-incubated with anti-hTLR5-IgA antibodies at a concentration of 10 µg/ml showed a reduction of 76% in IL-8 release.

HEK293 cells transfected with TLR5 (293-hTLR5 cells) secreted significant amounts of IL-8 after treatment with 100 ng/ml of flagella and flagellin of *C. sakazakii* (ST1). FliC from *S. enterica* serovar Typhimurium at 100 ng/ml showed an equal amount of IL-8 when compared to the flagella of *C. sakazakii* (Fig. 7). 293-hTLR5 cells pre-incubated with anti-hTLR5-IgA antibody at 10 µg/ml reduced in 76 and 74% the release of IL-8 in *C. sakazakii* flagella and flagellin, respectively; while, a reduction of 85% for FliC from *S. enterica* serovar Typhimurium was observed (Fig. 7).

## Discussion

All *Cronobacter* species are potential pathogens *in vivo*, especially toward neonates due to their vulnerable physiological state. However, only *C*. *sakazakii*, *C*. *malonaticus* and *C*. *turicensis* have been associated with neonatal deaths [35]. Currently, the genome sequences of many pathogenic bacteria have been described and pathogenomic studies allow us to compare sequences between different pathogens to identify specific or common virulence factors among them [36].

*Cronobacter* species adhered to HEp-2, Caco-2 and brain microvascular endothelial cells, producing two distinctive adherence patterns, a diffuse and a localized adhesion [17], [37]. Recently, it has been reported that the adherence levels to intestinal cells by *C*. *sakazakii* could be inhibited in the presence of prebiotic oligosaccharides [38]. However, the mechanism of binding to mammalian cells is not well-understood [17]. Two hypothetical proteins (ESA_00281 and ESA_00282) have been identified as possible adhesins involved in biofilm formation of *Cronobacter* species in abiotic material [28], [29]. Studies of comparative genome hybridization were undertaken on five other *C. sakazakii* strains, and representatives of the four other *Cronobacter* species (*C. malonaticus*, *C. muytjensii*, *C. dublinensis*, and *C. turicensis*). Among 4,382 annotated genes reviewed (*C. sakazakii* BAA-894), around 55% of the genes were common to all *C. sakazakii* strains and 43.3% were common to all five *Cronobacter* species, with a 10–17% absent genes. The majority of these genes are predicted to encode cellular essential functions such as energy metabolism, biosynthesis, DNA, RNA, protein synthesis, cell division and membrane transport. However, 5.1% of BAA-894 genes were absent in all *C. sakazakii* strains and 3.1% were absent in all *Cronobacter* species; these genes unique to *C. sakazakii* strains were in two separate clusters of proteins involved in pilus assembly, pilin FimA, porin PapC, and the chaperone PapD [35], [39]. In addition, no cluster or protein associated with flagella assembly was described in this study.

In this study, we demonstrate that flagella produced by *Cronobacter* species stimulate the activation of IL-8 and TNF-α in an infection model using human macrophages. The ability of flagella and flagellin of *Cronobacter* species to activate the release of pro-inflammatory cytokines has not been previously demonstrated, even though the activation of these molecules from other pathogens has been described. Pathogen-associated molecular patterns (PAMPs) such as LPS (Lipopolysaccharide), flagella, and pili are potent activators involved in the secretion of a variety of pro-inflammatory cytokines [34], [40], [41], [42]. Our data indicate that human macrophages incubated with purified flagella from *C. sakazakii* (ST1 and ST4), *C. malonaticus, C. muytjensii, C. turicensis*, and *C. dublinensis* contribute to the activation of significant levels of IL-8 and TNF-α in response to infection, while low levels of IL-10 were observed. According to other authors, it has been demonstrated that flagellin did not induce the production of IL-10 [32]. Recently, it has been reported that flagellins of H7, H8, H9, H11, and H21 serotypes from *E. coli* did not show differences in their ability to activate the release of IL-8 [43]. However, purified flagellins from STEC (Shiga like toxin) strains of different serotypes differed in their ability to induce IL-8 signaling suggesting that these flagellins vary in their ability to act as TLR agonists [43]. In addition, HCP (Hemorrhagic Coli Pilus) a type IV pilus, was recently described as an activator of IL-8 and TNF-α cytokines in intestinal cells [44]. The flagellins of *Salmonella* species [45], [46], [47] and other Gram-negative bacteria share similar inflammatory properties suggesting that some degree of sequence or structure conservation is responsible for the inflammatory response induction [48].

The diversity of the *Cronobacter* genus is well known as well as the variation among their pathogenic species [35], [39]. Recently, Hamby *et al.*, classified *Cronobacter* strains into two groups, pathogenic and non-pathogenic strains, observing an association between the source of the isolate, MLST type sequence (ST4) and inositol fermentation of the pathogenic strain [49]. The main sequence type identified in all strains of *Cronobacter* species analyzed by this method (383 isolated from *Cronobacter* distributed in 131 sequence types by MLST database) was ST4 corresponding to the *C. sakazakii* cluster [6], [12], [49]. Taking advantage of the diversity across the genus, we analyzed the ability of *C. sakazakii* sequence types ST1 and ST4 to induce anti- and pro-inflammatory cytokine release. The ST1 strain was employed in this study because it was the first sequenced genome of the *Cronobacter* species; our results, showed a high identity of 99% among purified flagellin (FliC) with the ESA_01288 protein described in the genome of this strain. This strain was isolated from powdered formula and it was associated with a clinical outbreak, while the ST4 strain was primarily associated with infant formula and meningitis infection [12]. Although the source of *C. sakazakii* ATCC 29004 (used in this study) is unknown, clustering of the dominance of the meningitis-related strains in a single ST (ST4) and inositol fermentation in this clustering, allowed for the association to a pathogenic isolate [6], [49]. Interestingly, our data showed no significant differences during the pro-inflammatory cytokine activation in macrophages in the presence of flagella from both strains (ST1 and ST4).

Flagellin as the main structural component of flagella, is considered the molecule responsible for triggering the mucosal inflammatory response by different enteric pathogens. Cytokine activation by several enteric flagellins is due to a high percentage of conserved domains between them. The flagellin monomer alone is capable of inducing cytokine’s release [50]. It remains to be determined what specific sequences of the flagellin protein contribute to the eukaryotic pro-inflammatory activity, demonstrated here. Based on our findings, we conclude that *Cronobacter* flagella are able to induce the release of a chemokine such as IL-8 in high levels and TNF-α I in less proportion compared to the EHEC EDL933 strain and *S. enterica* serovar Typhimurium flagella. Recently, other *Cronobacter* species sequence types have been associated with fatal cases of meningitis. In this study we demonstrated the ability of macrophages to release pro-inflammatory cytokines, a feature that allows a better understanding of the pathogenicity of this genus.

Likewise, secreted flagellin proteins (FlaA, FlaC and FlaD) from *V. cholerae* 395 identified by two-dimensional electrophoresis and mass spectrometry contribute to the inflammatory process [34]. It has been demonstrated that supernatants containing secreted flagellins FlaC and FlaD from the Δ*flaA* strain of *V. cholerae* 395 showed a 20-fold reduction in IL-8 release in comparison to the supernatant from the wild-type strain; and double isogenic mutants *flaAC* and *flaAD* were unable to trigger IL-8 release from HT-29 cells. These data demonstrated that FlaA is the main flagellin involved in IL-8 pro-inflammatory release [34]. A dose-dependent inhibition was observed using rabbit undiluted and diluted (1∶10, 1∶100, and 1∶200) anti-flagella antibodies when directed against purified flagella. Interestingly, we observed a striking inhibition in its capability to trigger IL-8 release when the undiluted antibody and a 1∶10 dilution was used, producing a significant reduction of 94 and 88%, respectively. In the case of TNF-α and IL-10 release, activation was completely abolished when any of the three dilutions of the antibody against flagella were employed. The ability to activate the release of IL-8 and TNF-α was also evaluated when flagella were dissociated into flagellin monomers, the same patterns were observed in assays using flagella. These results demonstrated that flagella in their native and dissociated conformations did not show differences in their ability to release IL-8 and TNF-α because the same percentage of inhibition by addition of the polyclonal anti-flagella antibody to the flagellin was observed.

The innate immune system employs intracellular and extracellular pattern recognition receptors (PRRs) to detect microbial products through the identification of PAMPs. Families of PRRS, Toll-like receptors (TLRs), and NOD-like receptors (NLRs) are important elements in triggering an innate immune response, such as TLR5 (extracellular) and NLRC4 (intracellular), two receptors that are recognized by flagellins [51], [52]. In this study, anti-hTLR5-IgA antibodies were also used for functional blocking of flagellin stimulation by TLR5 activation. The activation of TLR5 produced a signaling cascade, triggering the activation of the nuclear factor-κβ (NF-κβ). The initiation of this pathway induces the transcription of a variety of host defense genes, including IL-8, IL-6, TNF-α and IL-1 [53]. Our data indicated that IL-8 and TNF-α release in macrophages is dependent upon a flagellin-TLR5 interaction. TNF-α and IL-10 release was inhibited when 20 µg/ml of the anti-hTLR5-IgA antibody were used; while IL-8 showed a decrease of 59% with 100 ng/ml of flagellar protein and 53% with flagellin. Similar data were observed for FliC from *S. enterica* serovar Typhimurium, with a 60% decrease. Flagellin secreted by many bacterial pathogens has been shown to be a potent activator of pro-inflammatory cytokines via TLR5 in epithelial cells [33], [54]. To provide TLR5 signaling by flagella and flagellin from *Cronobacter* species, we performed a transfection assay using 293-hTLR5 cells. 293-hTLR5 cells were obtained by stable transfection of HEK293 cells with pUNO-hTLR5 [55]. Our results showed TLR5 stimulation by quantification of IL-8 when flagella and flagellin were tested, and a significant decrease of IL-8 was observed when the anti-hTLR5-IgA antibody was used. Similar results were observed with flagella from *Burkholderia cenocepacia* and *Bordetella bronchiseptica* [56], [57]. This data support that flagella and flagellin from *Cronobacter* species are recognized by TLR5 for the activation of pro-inflammatory cytokines. However, intracellular receptors in the NLRC4 inflammasome respond to bacterial flagellin and other components such as types III and IV secretion systems. This receptor has been well studied in mice infected with *Legionella pneumophila*, *S. enterica* serovar Typhimurium and/or *Pseudomonas aeruginosa* [58], [59]. In this study, the extracellular TLR5 receptor was tested; however, it is necessary to perform other assays to demonstrate whether *Cronobacter* flagella are involved in the signaling through the intracellular receptor NCLR4.

In conclusion, flagella and flagellin from *C. sakazakii* (ST1 and ST4), *C. malonaticus*, *C. muytjensii*, *C. turicensis*, and *C. dublinensis* elicit a strong inflammatory response in human macrophages, including the production of neutrophil chemoattractant IL-8 and TNF-α. The activation of these cytokines is due to the TLR5-induced signaling by flagella, which provides a better understanding of the pathogenesis of these bacteria. Further *in vivo* characterization of the mechanisms by which flagella from *Cronobacter* species signal extracellular and intracellular receptors will be necessary to demonstrate the role of flagella in host innate immune response.

## Materials and Methods

### Bacterial Strains and Growth Conditions

*C. sakazakii* ATCC BAA-894 ST1 [(sequence type 1 strain for MLST) genome sequence, Genebank:CP000785] and *C. sakazakii* ATCC 29004 ST4 [(sequence type 4 strain for MLST)appears to be a stable clone with clinical significance in meningitis infections] sequence type classification was reported by Baldwin *et al.* [11]. *C. malonaticus* CDC 1058-77^T^ (type strain), *C. muytjensii* ATCC 51329^T^ (type strain), *C. turicensis* LMG 23827^T^ (type strain) genome sequence (FN543093 to FN543096), and *C. dublinensis* LMG 23823^T^ (type strain), donated by Carol Iversen (Nestlé Research Center, Lausanne, Switzerland) were cultured on Trypticase Soy Agar (TSA) (BD, Difco, New Jersey, USA) at 37°C. Flagella from EHEC strain EDL933 and *Salmonella enterica* serovar Typhimurium were used as positive controls. Purified flagella from *C. sakazakii* were used for the generation of polyclonal antibodies by immunization of rabbits as described below.

### Purification of Flagella

*C. sakazakii* ATCC BAA-894 ST1, *C. sakazakii* ATCC 29004 ST4, *C. malonaticus, C. muytjensii, C. turicensis*, and *C. dublinensis* grown on TSA agar were cultured and resuspended in PBS (phosphate-buffered saline, pH 7.4). Bacteria were separated twice by centrifugation at 8,000 *g* for 20 min and the supernatant was centrifuged at 10,000 *g* for 30 min to remove outer membranes and bacterial debris. The flagella-containing supernatant was precipitated adding solid ammonium sulfate up to 50% saturation. Flagella were recovered by centrifugation at 8,000 *g* for 30 min and extensively dialyzed against PBS three times [34]. Protein concentration was determined with the Bradford method [60].

### Transmission Electron Microscopy (TEM) and Immunogold Labeling

*Cronobacter* species were grown in Tripticase Soy Broth (TSB) overnight at 37°C. The bacterial culture was mounted on a formvar-carbon-coated grid for 5 min and negatively stained with 1% sodium-phosphotungstic acid (pH 7.2) for 5 min. Immediately, the samples were washed three times with distilled water and visualized under a Philips transmission electron microscope (TEM), as previously described [61]. For immunogold assays, the bacterial cultures were mounted directly on formvar-carbon-coated grids and labeled using polyclonal anti-flagella polyclonal antibodies for 2 h. After washing three times with distilled water, bacteria were incubated with a goat anti-rabbit IgG labeled with 10-nm gold particles (Sigma, Aldrich) for 2 h, stained with 1% sodium-phosphotungstic acid for 5 min and visualized by TEM. The antibodies were diluted 1∶10 in PBS containing 1% (wt/vol) bovine serum albumin (BSA).

### Denaturing Sodium Dodecyl-sulfate Polyacrylamide Gel Electrophoresis (SDS-PAGE)

Purified flagellins were visualized by SDS-PAGE on 12% acrylamide gels and stained with Coomassie blue [62]. Previously, purified flagella were resuspended in sample buffer 4X, heated at boiling point for 5 min, and loaded onto 12% polyacrylamide gels. Flagellins were visualized after staining the gels with a 0.25% Coomassie blue solution.

### Protein Sequencing

The protein bands of interest were excised from a Coomassie Brilliant Blue R-250-stained gel digested with trypsin and identified by mass spectrometry (3200 QTRAP hybrid tandem mass spectrometer, Applied Biosystem/MDS Sciex, Concord, ON, Canda). LC/MS/MS analysis of tryptic peptides was carried out using a NanoAcquity ultraperformance liquid chromatograph (UPLC) (Waters Corporation) coupled to a Q-ToF Synapt High Definition Mass Spectrometer (Waters Corporation) equipped with a NanoLockSpray ion source.

### Flagella Polyclonal Antisera

All experiments with animals were performed in accordance with Mexican Official Standard NOM-062-ZOO-1999. For the generation of polyclonal antibodies against the flagella fraction of *C. sakazakii*, one New Zealand white rabbit (2.0–2.5 kg) received subcutaneous injections of flagella (0.75 mg in 0.5 ml of PBS emulsified with an equal volume of Freund’s adjuvant). The animal received booster injections (on day 14 and 22 after initial priming immunization) with 0.35 mg of purified flagella in 0.5 ml of PBS with 0.5 ml of incomplete Freund’s adjuvant. Antibody titers were examined by Western-blot and ELISA at 0, 2, 3 and 4 weeks after the initial immunization and the animals were killed by cardiac exsanguination at the end of the fourth week, and the serum collected was stored at −20°C until use.

Immunoglobulin G (IgG) was obtained from sera of rabbits immunized with flagella that showed high reactivity to these proteins. Briefly, 250 ml of Protein G-agarose (Invitrogene, USA) were mixed with 500 ml of each sera, incubated for 20 min at room temperature (RT) mixing gently every 2 min, centrifuged for 30 s at 500×*g* (Sorvall RC5), the supernatant was discarded and the IgG-Protein G-agarose complex washed five times with 0.1 M glycine, pH 9.0. After the last wash, the complex was dispersed in 750 ml of elution buffer (0.1 M glycine–HCl, pH 2.2) and centrifuged at 500 *g* for 30 s. The supernatant containing the purified IgG fraction was adjusted to pH 7.0 with 2 M Tris base. Protein concentration was determined by Bradford procedure [60].

### Immunodetection

Purified proteins were resolved by SDS-PAGE gels and transferred onto a PVDF membrane for 2 h at 90 volts. The immobilized proteins were incubated for 1 h with a primary antibody against flagella at a 1∶10,000 dilution and washed three times with PBS. Immediately, the membranes were incubated for 1 h with a goat anti-rabbit IgG secondary antibody conjugated to peroxidase (1∶10,000 dilution) (Sigma-Aldrich) and washed three times with PBS. The blots were developed with Immobilon Western (Millipore).

### Peripheral-blood Monocyte-derived Macrophage (MDM) Preparation and Culture

Mononuclear cells were separated from four healthy donor buffy coats by gradient centrifugation with Lymphoprep (Nycomed AS, Oslo, Norway). Monocytes were then isolated by magnetic depletion of T cells, NK cells, B cells, dendritic cells, and basophils, using a cocktail of anti-CD3, anti-CD7, anti-CD19, anti-CD45RA, anti-CD56, and anti-IgE antibodies (Miltenyi Biotec, Bergisch Gladbach, Germany). Macrophages derived from human monocytes were obtained from a Buffy Coat given by the Blood Bank from the Hospital Infantil de México. A serological analysis with specific markers was performed to choose a healthy human donor. Buffy coats from patients were randomly selected by the people working in the Blood Bank. Purity (≥95%) was assessed by flow cytometry using fluorescein isothiocyanate (FITC)-conjugated anti-CD14 antibody (Pharmingen, San Diego, CA, USA). Monocytes were differentiated from a 1×10^5^ macrophages/ml solution when cultured for seven days at 37°C and 5% CO_2_ in ultra-low-adherence 24-well culture plates (Costar, Corning, NY, USA), using RPMI-1640 medium supplemented with 10% fetal calf serum (Gibco, Invitrogen USA). The cells were washed three times, incubated with different protein concentrations of flagella (1, 10, 25, 50, and 100 ng/ml) in 24-well culture plates and without proteins as a negative control. *Escherichia coli* O111:B4 lipopolysaccharide (LPS) (Sigma Chemical Co., St. Louis, MO, USA) and purified flagellin from EHEC strain O157:H7 and *S. enterica* serovar Typhimurium were used as positive controls at concentrations of 100 ng/ml, respectively. All cultures were incubated for 12 and 24 h and were tested by triplicate in three different experiments.

### Quantification of Anti- and Pro-inflammatory Cytokines by ELISA

A sandwich ELISA using 96-well plates coated overnight at 4°C, and using a concentration of 10 µg/ml goat anti-human IL-8, TNF-α and IL-10 antibodies (BD Biosciences Pharmingen, San Diego, CA, USA), was performed as previously described [54]. The supernatants obtained after 12 and 24 h incubation of flagella with the macrophages, were added to the wells with rabbit anti-human IL-8, TNF-α and IL-10. Previously, cytokines’ concentrations were quantified using an ELISA kit (BD Pharmingen, San Diego, CA, USA). In different experiments, levels of IL-8, TNF-α and IL-10 were measured in supernatants with 1, 10, 25, 50, and 100 ng/ml of flagella. The complexes were detected with goat anti-rabbit IgG peroxidase conjugate (Sigma-Aldrich, St. Louis, MO, USA) and peroxidase substrate. Color development was read at an absorbance of 450 nm. In addition, purified flagella were dissociated in monomers at 65°C for 2 h, to evaluate their ability to release IL-8, TNF-α and IL-10 in macrophages.

### Removal of Endotoxin from *Cronobacter* Species Protein using Polymixin B

Endotoxin in *Cronobacter* species flagella protein was removed using polymixin B agarose (Sigma, St Louis CA) according to manufacturer’s recommendation. Briefly, aliquots of 0.5 ml of polymixin B agarose were mounted into Poly-prep disposable columns (Pierce) and equilibrated in PBS. Columns were washed with 5 volumes of 1% sodium deoxycholate followed by 10 volumes of PBS. 250 µl of *Cronobacter* species protein at 100 µg/ml were loaded onto 0.5 ml Detoxi columns and incubated at room temperature for 60 min. The columns were eluted with PBS and 250 µl fractions were collected. The fractions obtained were analyzed in SDS-PAGE gels after staining with Coomassie blue.

### Measurement of Endotoxin Activity

The endotoxin activity of purified flagella of *Cronobacter* species was assayed using the LAL assay (Lonza), according to manufactureŕs recommendation. Briefly, 100 µl of protein was incubated with 100 µl of LAL for 60 min at 37°C by duplicate. After 1 h incubation, the samples were analyzed visually by simply inverting the reaction tube. In the presence of endotoxin, gelation occurs whereas in the absence of endotoxin, gelation does not occur.

### Transient Expression of TLR5 in HEK293 Cells (293-hTLR5)

Human epithelial kidney cells (HEK293-hTLR5) were obtained from InvivoGen (San Diego, CA) and grown in Dulbecco modified Eagle medium (DMEM) high glucose supplemented with 10% fetal calf serum (Gibco, Invitrogen USA) and 10 µg/ml blasticidine (InvivoGen). HEK293-hTLR5 cells were removed with PBS after reached a 70–80% confluence and seeded 1.5×10^5^ cells per well. The plate was incubated at 37°C with 5% CO_2_ and by 18–20 h. The medium was removed and replaced with fresh growth medium DMEM high glucose supplemented with 10% fetal calf serum and 10 µg/ml blasticidine [56]. The cells were stimulated by 24 h with 100 ng/ml of flagella and flagellin of *C. sakazakii* (ST1) and FliC from *S. enterica* serovar Typhimurium, respectively. Blocked assay was performed using 10 µg/ml of anti-hTLR5-IgA monoclonal antibodies for each flagella tested. TLR5 stimulation was assessed determining the levels of IL-8 using an ELISA kit, as previously described.

### Statistical Analyses

All statistical analyses were performed using the SPSS software (SPSS Inc. Chicago IL) Pearson’s correlation coefficients between the levels of cytokine secretion and those of *Cronobacter* species flagella and flagellin were calculated. The significance of the differences between each cytokine secretion levels induced by different treatments was calculated using one-way analysis of variance, followed by Sheffe posthoc test. The *p* value was determined using a tow-tails test.

### Study Approval

The study was approved by the Research, Ethics and Biosecurity Committee of the Hospital Infantil de México with permit number HIM/2011/082.
