# Correction: Flagella from Five *Cronobacter* Species Induce Pro-Inflammatory Cytokines in Macrophage Derivatives from Human Monocytes

**DOI:** 10.1371/annotation/30b5bfd2-aa2a-49bc-9af5-14ff8997eee3

**Published:** 2013-05-24

**Authors:** Ariadnna Cruz-Córdova, Luz M. Rocha-Ramírez, Sara A. Ochoa, Bertha Gónzalez-Pedrajo, Norma Espinosa, Carlos Eslava, Ulises Hernández-Chiñas, Guillermo Mendoza-Hernández, Alejandra Rodríguez-Leviz, Pedro Valencia-Mayoral, Stanislaw Sadowinski-Pine, Rigoberto Hernández-Castro, Iris Estrada-García, Onofre Muñoz-Hernández, Irma Rosas, Juan Xicohtencatl-Cortes

There was an error in name of the fourth author.

The correct name is: Bertha González-Pedrajo

The correct Citation is: Cruz-Córdova A, Rocha-Ramírez LM, Ochoa SA, González-Pedrajo B, Espinosa N, et al. (2012) Flagella from Five Cronobacter Species Induce Pro-Inflammatory Cytokines in Macrophage Derivatives from Human Monocytes. PLoS ONE 7(12): e52091. doi:10.1371/journal.pone.0052091 

